# The burrower bug *Macroscytus japonensis* (Hemiptera: Cydnidae) acquires obligate symbiotic bacteria from the environment

**DOI:** 10.1186/s40851-024-00238-9

**Published:** 2024-08-02

**Authors:** Takuma Nakawaki, Shuto Watanabe, Takahiro Hosokawa

**Affiliations:** 1 Graduate School of Systems Life Sciences, Kyushu University, Fukuoka, 819-0395 Japan; 2 Faculty of Science, Kyushu University, Fukuoka, 819-0395 Japan

**Keywords:** Heteroptera, Pentatomoidea, Gut symbiont, γ-Proteobacteria, Molecular phylogeny, Symbiont cultivability, Natural population

## Abstract

Many plant-feeding stinkbugs belonging to the infraorder Pentatomomorpha possess a specialized symbiotic organ at the posterior end of the midgut, in which mutualistic bacterial symbionts are harbored extracellularly. In species of the superfamily Pentatomoidea, these symbionts typically are verticallytransmitted from host mothers to offspring, whereas in species of the superfamilies Coreoidea and Lygaeoidea they are acquired from the environment. In the pentatomoid family Cydnidae, vertical symbiont transmission has been reported in several species. Here, we report the first case of environmental symbiont acquisition in Cydnidae, observed in the burrower bug *Macroscytus japonensis*. A comprehensive survey of 72 insect samples from 23 sites across the Japanese archipelago revealed that (1) symbionts exhibit remarkably high diversity, forming six distinct phylogenetic groups within the Enterobacteriaceae of the γ-Proteobacteria, (2) most symbionts are cultivable and closely related to free-living *Pantoea*-allied bacteria, and (3) symbiont phylogenetic groups do not reflect the host phylogeny. Microbial inspection of eggs revealed the absence of bacteria on the egg surface. These results strongly suggest that symbionts are acquired from the environment, not vertical transmission. Rearing experiments confirmed environmental symbiont acquisition. When environmental symbiont sources were experimentally withheld, nymphs became aposymbiotic and died before molting to the second instar, indicating that nymphs environmentally acquire symbionts during the first-instar stage and that symbionts are essential for nymphal growth and survival. This study highlights Cydnidae as the only pentatomoid family that includes species that environmentally acquire symbionts and those that vertically transmit symbionts, providing an ideal platform for comparative studies of the ecological and environmental factors that influence the evolution of symbiont transmission modes.

**Supplementary Information:**

The online version contains supplementary material available at 10.1186/s40851-024-00238-9.

## Background

Diverse insects that feed on nutrient-limited diets, such as plant sap, vertebrate blood, and woody materials, harbor mutualistic symbiotic microorganisms that play important biological roles, including nutrient provision and food digestion [1–4]. These symbionts are present on the body surface, in the alimentary tract, within the body cavity, or even inside the cells of host insects [5, 6]. For example, the symbiotic bacterium *Buchnera aphidicola*, which resides in the bacteriomes of aphids, provides essential amino acids to the host insects [7]. Similarly, the symbiotic bacterium *Stammera capleta*, which is present in the foregut symbiotic organs of cassidine tortoise beetles, upgrades the digestive capacity of the host insects [8].

Plant-feeding stinkbugs of the superfamilies Pentatomoidea, Coreoidea and Lygaeoidea, all of which belong to the infraorder Pentatomomorpha (Insecta: Hemiptera), generally have mutualistic associations with gut symbiotic bacteria. These stinkbugs possess numerous sac-like outgrowths, called crypts, along the posterior region of the midgut (referred to as the midgut fourth section), where specific symbiotic bacteria are harbored extracellularly [1, 9–13]. When symbionts are experimentally withheld, such aposymbiotic stinkbugs suffer substantial fitness defects, including retarded growth, elevated mortality, morphological abnormalities, reduced offspring, and/or complete sterility [1, 14–45], indicating the important biological roles of symbionts in their hosts.

In the superfamily Pentatomoidea, gut symbiotic bacteria are usually members of the γ-Proteobacteria and are vertically transmitted from host mothers to offspring via egg surface contamination or symbiont-containing materials deposited near eggs or young nymphs. This mode of symbiont transmission has been demonstrated in families such as Pentatomidae [15, 18, 21–23, 26, 29, 32–37, 39, 40], Scutelleridae [28, 38, 46], Acanthosomatidae [20], Plataspidae [16, 17], Urostylididae [31], Parastrachiidae [25], and Cydnidae [14, 27, 47]. These vertically transmitted symbionts are typically uncultivable, probably due to their adaptation to the intrahost environment. The only known exception among pentatomoid stinkbugs is the saw-toothed stinkbug *Megymenum gracilicorne* of the family Dinidoridae, in which cultivable γ-proteobacterial symbionts are not vertically transmitted, but acquired from environmental sources [43]. In the superfamilies Coreoidea and Lygaeoidea, gut symbiotic bacteria generally belong to the β-Proteobacteria and are acquired environmentally. This mode of symbiont transmission has been demonstrated for families such as Alydidae [19, 30, 48], Coreidae [41, 44, 45, 49], Blissidae [24], and Berytidae [42], with a mixed mode of environmental and vertical transmission documented in one blissid species [50]. These environmentally acquired symbiotic bacteria are often cultivable and can be isolated from both the environment and the midgut crypts of stinkbugs. Given the interest of evolutionary biologists in the ecological and environmental factors influencing symbiont transmission modes (vertical, environmental, or mixed) [51–53], pentatomomorphan stinkbugs, which exhibit a variety of transmission modes, represent a fascinating insect group.

**Fig. 1:**
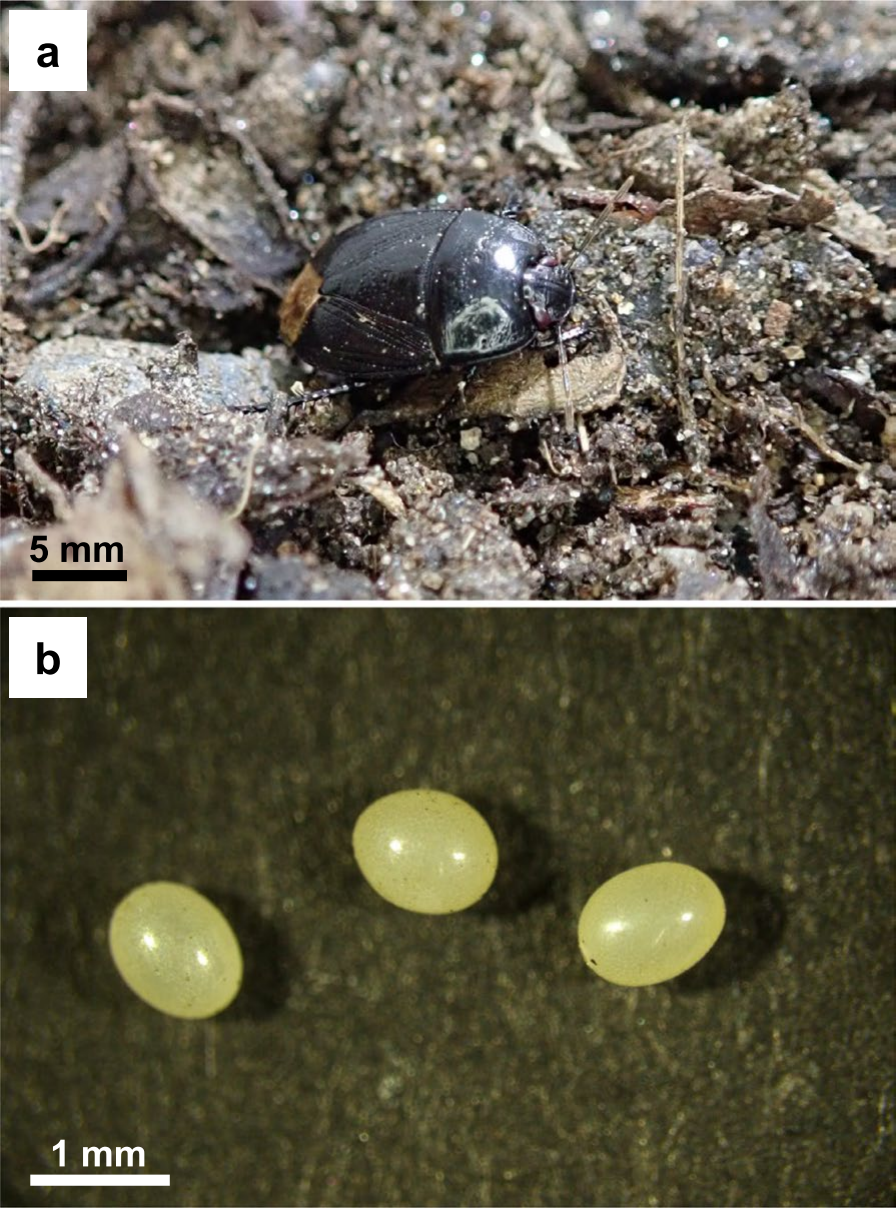
Adult insect (**a)** and eggs (**b**) of *M. japonensis*

The pentatomoid family Cydnidae comprises over 144 genera and at least 1,185 species worldwide, many of which are known as “burrower bugs” due to their fossorial lifestyle, sucking plant roots and seeds [54]. Vertical symbiont transmission has been observed in four cydnid species, *Cydnus atterimus* (= *Brachypelta atterima*), *Canthophorus niveimarginatus*, *Adomerus triguttulus*, and *A. rotundus* [14, 27, 47], with no documented cases of environmental symbiont acquisition in this insect group. The burrower bug *Macroscytus japonensis* (Fig. 1A) is widely distributed in eastern and southeastern Asian countries, including Japan [55]. Specific γ-proteobacterial gut symbionts have been identified in two Japanese populations, but they are distantly related to the vertically transmitted symbionts of *C. niveimarginatus*, *A. triguttulus*, and *A. rotundus* [56], suggesting the possibility of environmental symbiont acquisition in *M. japonensis*. If confirmed, the family Cydnidae would encompass species that vertically transmit symbionts as well as those that environmentally acquire symbionts, making it a more compact and tractable insect group for investigating the evolution of symbiont transmission modes. The present study, based on an extensive and detailed survey of field samples and rearing experiments, reports findings on environmental symbiont acquisition, as well as symbiont diversity, cultivability, and their essential role in host growth and survival in *M. japonensis*.

## Materials and methods

### Insect samples

We collected adult *M. japonensis* from leaf litter or by light trapping at 23 sites in Japan (Fig. S1). The insect samples used for the analysis of symbiont diversity and cultivability are listed in Table S1. The insect samples used in the egg inspection and rearing experiments were collected in Itoshima, Fukuoka, Japan in 2022. All insect samples were brought to the laboratory and maintained at 25 °C under a long-day regimen (16 h light, 8 h dark) in sterilized Petri dishes (90 mm in diameter, 20 mm in height). These dishes were provided with autoclaved peanuts and almonds as food [57] and autoclaved sand moistened with sterilized water as shelter.

### Symbiont cultivation and DNA preparation

Cultivability tests of midgut symbiotic bacteria were performed as previously described for other pentatomoid stinkbugs with some modifications [35, 43]. The midgut fourth section was dissected from each adult insect in sterilized phosphate-buffered saline (PBS) (FUJIFILM Wako Pure Chemical) using fine forceps under a dissection microscope. The tissue was homogenized in 500 µL of PBS in a plastic tube using a plastic pestle. Subsequently, 20 µL of the suspension was spread on an LB agar plate and incubated at 26 °C for 24 h. If colonies formed, they were subjected to DNA extraction using the NucleoSpin Tissue Kit (Macherey–Nagel), and the bacterial isolates were stored as glycerol stocks at –80 °C. The remaining suspension was centrifuged at 13,000 rpm for 1 min, and the precipitated bacterial cells and insect tissue fragments were subjected to DNA extraction.

### PCR, cloning, sequencing and molecular phylogenetic analyses

Bacterial genes were amplified from the DNA samples by PCR using AmpliTaq 360 DNA Polymerase (Applied Biosystems) and primers 16SA1 (5′- AGA GTT TGA TCM TGG CTC AG-3’) and 16SB1 (5’-TAC GGY TAC CTT GTT ACG ACT T-3′) for the 16S rRNA gene [58], and PANgroELF (5′-TCG ARC TGG AAG ACA AGT TCG-3′) and PANgroELR (5’-CTT CTT CGA TYT GCT GAC G -3′) for the *groEL* gene. The PCR products (1.5 kb for the16S rRNA gene and 0.9 kb for the *groEL* gene) were cloned and sequenced as described previously [20]. The insect mitochondrial *cytochrome oxidase I* (*COI*) gene was also amplified using the primers LCO1490 (5′-GGT CAA CAA ATC ATA AAG ATA TTG G-3′) and HCO2198 (5′-TAA ACT TCA GGG TGA CCA AAA AAT CA-3′) [59] and subjected to direct sequencing. The *COI* gene of the closely related congeneric species *M. fraterculus* was also sequenced for use as an outgroup in phylogenetic analysis (see Table S1 for sample information). The accession numbers of the nucleotide sequences determined in this study are listed in Table S1 and Table S2. Multiple alignments of nucleotide sequences were generated using the program MUSCLE [60], from which gap-containing sites were removed. Substitution models were selected, and maximum-likelihood phylogenies were constructed using the program MEGA 7.0.26 [61].

### Egg inspection

Unlike other pentatomoid stinkbugs, females of some cydnid species, including *M. japonensis*, do not form egg masses, but lay their eggs individually [62] (Fig. 1B). Six *M. japonensis* females were allowed to lay eggs in separate rearing containers. From each container, six eggs were collected, rinsed with sterile water to remove sand, individually crushed in a plastic tube using a plastic pestle, and subjected to DNA extraction. Diagnostic PCR using primers 16SA1 and 16SB1 for the bacterial 16S rRNA gene was performed to detect the presence of symbiotic bacteria on the egg surface. The quality of the template DNA samples was verified by PCR amplification of the insect mitochondrial *COI* gene. The DNA sample extracted from the midgut fourth section from an adult female (ITSM1) was used as a positive control for these PCRs.

### Rearing experiment to confirm environmental symbiont acquisition

An isolated and preserved strain of the gut symbiont from an adult female (ITSM1; see Table S1 for sample information) was cultured in LB liquid medium at 26 °C and diluted to 10^7^–10^8^ CFU mL^–1^ in sterile water. Soil samples were collected from a site in Itoshima, Fukuoka, Japan, where *M. japonensis* is commonly found. About 200 eggs were collected from rearing containers with field-collected adults, rinsed with sterile water, and placed in sterilized plastic Petri dishes (60 mm in diameter, 15 mm in height) with 3–8 eggs per dish. Each Petri dish was assigned to one of the following experimental treatments: (1) sterile water treatment, (2) symbiont-suspended water treatment, or (3) field-collected soil treatment. In the sterile water treatment, food (autoclaved peanuts and almonds) and a piece of cotton soaked with sterile water were provided to each Petri dish (Fig. S2a). In the symbiont-suspended water treatment, food and a piece of cotton soaked with symbiont-suspended water, prepared as described above, were provided to each Petri dish (Fig. S2b). In the field-collected soil treatment, food and approximately 5 g of the soil sample soaked with sterile water were provided to each Petri dish (Fig. S2c). In all treatments, the eggs were incubated, and hatched nymphs (63 in sterile water treatment, 51 in symbiont-suspended water treatment, and 50 in field-collected soil treatment) were reared to the third-instar stage. Third-instar nymphs were then transferred to new sterile dishes (90 mm in diameter, 20 mm in height) and provided with food and a piece of cotton soaked with sterile water until all nymphs reached adulthood or died. All newly emerged adults were subjected to dissection of the midgut fourth section and DNA extraction. The 16S rRNA gene of the gut symbiotic bacteria was amplified, cloned, and sequenced as described above.

## Results

### Diversity of gut symbiotic *bacteria*

**Fig. 2:**
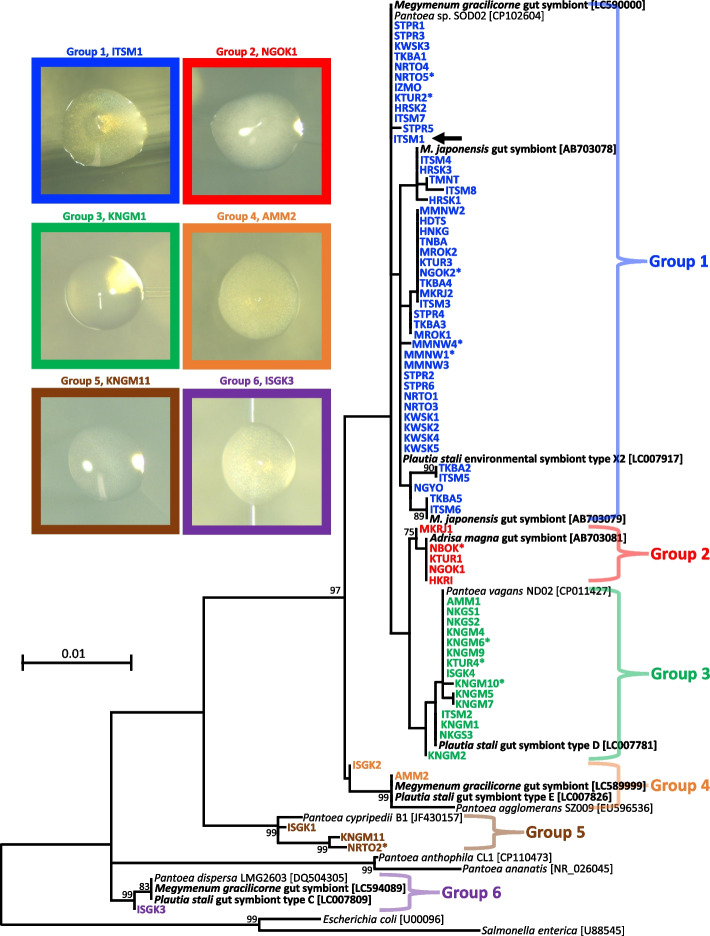
Phylogenetic placement of gut symbiotic bacteria from field-collected *M. japonensis* adults based on 16S rRNA gene sequences. A maximum likelihood tree inferred from 1,413 aligned nucleotide sites is shown with bootstrap values of no less than 70%. The gut symbiotic bacteria of *M. japonensis* are colored, and the sample IDs are listed in Table S1. Asterisks denote gut symbiotic bacteria uncultivable on LB agar plates. The arrow indicates the isolated bacterial strain used in the rearing experiment. The gut symbiotic bacteria of other stinkbug species are highlighted in boldface. Brackets contain sequence accession numbers. Photos show the colony forms of gut symbiotic bacteria from each insect sample on LB agar plates

We examined 72 insect samples of *M. japonensis* from 23 sites across the Japanese archipelago (see Table S1 and Fig. S1). The midgut fourth sections from these samples were subjected to DNA extraction, followed by PCR amplification and cloning of the bacterial 16S rRNA and *groEL* genes. For each gene, 3–5 clones per insect were sequenced, all of which yielded identical nucleotide sequences, suggesting that many of these bugs were likely colonized by a single predominant strain. Phylogenetic analysis of the 16S rRNA gene sequences revealed a remarkably high diversity of *M. japonensis* gut symbionts within the Enterobacteriaceae of the γ-Proteobacteria (Fig. 2). Phylogenetic analysis of the *groEL* gene sequences also showed results largely consistent with those obtained from the 16S rRNA gene sequences (Fig. S3). These phylogenetic analyses revealed that the symbiotic bacteria of *M. japonensis* and other related *Pantoea*-allied bacteria formed six distinct phylogenetic groups (hereafter referred to as groups 1–6). Note that the bootstrap support for some of these groups was low in the 16S rRNA phylogeny (Fig. 2), but significantly high (> 98% each) in the *groEL* phylogeny (Fig. S3). Group 1, the largest clade, included gut symbionts from 63.9% (46/72) of the *M. japonensis* samples. It also included previously reported gut symbionts of *M. japonensis* [56], the cultivable gut symbiont of the dinidorid stinkbug *Me. gracilicorne* [43], an environmental bacterium, designated type X2, which was shown to be capable of symbiosis with the pentatomid stinkbug *Plautia stali* [35], and *Pantoea* sp. SOD02, a bacterial strain isolated from an urban freshwater stream [63]. Group 2 consisted of gut symbionts from 6.9% (5/72) of the *M. japonensis* samples and the gut symbiont of the cydnid stinkbug *Adrisa magna* [56]. Group 3 comprised gut symbionts from 20.8% (15/72) of the *M. japonensis* samples, the cultivable gut symbiont, designated type D, of the pentatomid stinkbug *P. stali* [35], and *Pantoea vagans* ND02, a bacterial strain isolated from a waterfall (GenBank accession number CP011427). Group 4 comprised gut symbionts from 2.8% (2/72) of the *M. japonensis* samples, the cultivable gut symbiont of the dinidorid stinkbug *Me. gracilicorne* [43], the cultivable gut symbiont, designated type E, of the pentatomid stinkbug *P. stali* [35], and *Pantoea agglomerans* SZ009, a bacterium isolated from the surface of mangrove roots (GenBank accession number EU596536). Group 5 consisted of gut symbionts from 4.2% (3/72) of the *M. japonensis* samples and *Pantoea cypripedii* B1 and NE1, bacterial strains isolated from the rhizosphere of leguminous plants (GenBank accession numbers JF430157 and CP024768). Group 6 consisted of gut symbionts from 1.4% (1/72) of the *M. japonensis* samples, the cultivable gut symbiont of the dinidorid stinkbug *Me. gracilicorne* [43], the cultivable gut symbiont, designated as type C, of the pentatomid stinkbug *P. stali* [35], and *Pantoea dispersa* LMG2603, a bacterial strain isolated from the soil (GenBank accession numbers DQ504305 and LC007455). Mapping of the symbiont groups onto the mitochondrial phylogeny of the host revealed that the symbiont groups did not reflect the host genotypes, although group 1 and 3 symbionts tended to be located in specific mitochondrial clades (Fig. S4a). However, we did identify a geographic difference in the infection frequency of group 1–6 symbionts between mainland and southwestern island populations. In mainland populations, group 1 symbiont infections were most prevalent (85.2%) and groups 4 and 5 symbiont infections were absent, whereas in southwest island populations, in groups 1 and 2 symbiont infections were absent, while in group 3 symbiont infections were most prevalent (72.2%) (Fig. S4b).

### Cultivability of gut symbiotic *bacteria*

After the homogenate of dissected midgut fourth sections was plated, bacterial colonies formed within 24 h in most *M. japonensis* samples. One to three colonies per insect sample were subjected to 16S rRNA gene sequencing and compared to the symbiont sequence derived from the midgut fourth section of the respective insect sample. In 86.1% (62/72) of the insect samples, the colony-derived sequences exhibited 100% identity with the midgut-derived symbiont sequence, indicating the cultivability of the gut symbiotic bacteria (Table S1). Symbiont cultivability was not dependent on symbiont phylogenetic group (Fig. 2 and Fig. S3).

### No superficial bacterial contamination of eggs

When 36 eggs from six *M. japonensis* females were individually subjected to diagnostic PCR using universal bacterial primers, no bacteria were detected in 34 eggs, whereas a faint band was observed in two eggs after 35 cycles of PCR (Fig. S5).

### Confirmation of environmental symbiont acquisition

**Fig. 3:**
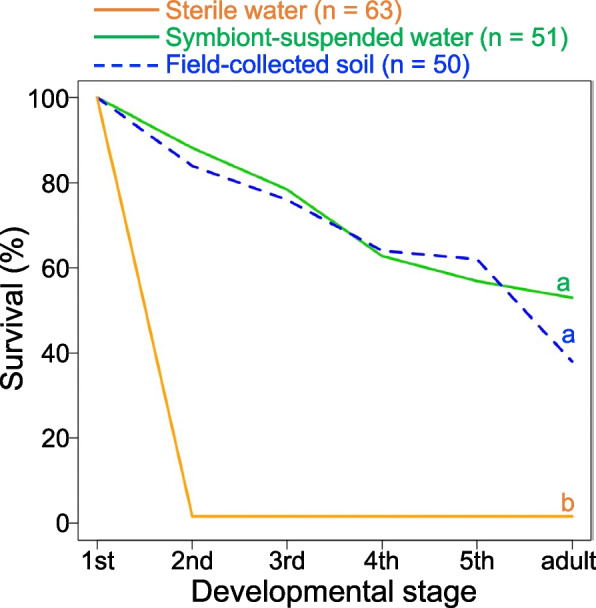
Survival of *M. japonensis* nymphs in the rearing experiment. The orange line represents nymphs supplied with food and sterile water; the green line represents nymphs supplied with food and symbiont-suspended water; and the blue dotted line represents nymphs supplied with food and field-collected soil. Letters indicate significant differences in nymphal survival to adult emergence (*P* < 0.0001, log-rank test with Bonferroni correction)

Figure 3 shows the survival curves from the first-instar stage to the adult stage in the three rearing experiment treatments. When newborn nymphs were reared under sterile conditions, only 1.6% (1/63) reached adulthood, with 98.4% (62/63) dying at the first instar. In contrast, when eggs and newborn nymphs were supplied with symbiont-suspended water, 52.9% (27/51) reached adulthood. Similarly, when eggs and newborn nymphs were provided with field-collected soil, 38.0% (19/50) reached adulthood. Survival to adult emergence was significantly greater in both the symbiont-suspended water and field-collected soil treatments than in the sterile water treatment (both *P* < 0.0001, log-rank test with Bonferroni correction). There was no statistically significant difference in nymphal survival between the symbiont-suspended water and field-collected soil treatment groups.

**Fig. 4:**
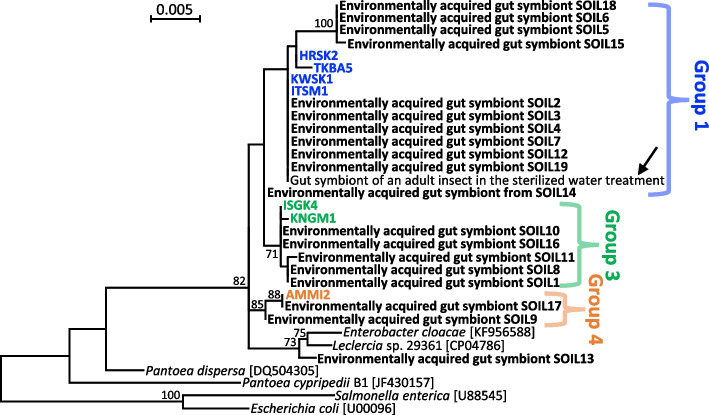
Phylogenetic placement of gut symbiotic bacteria associated with *M. japonensis* adults that emerged after nymphal incubation with field-collected soil. A maximum likelihood tree inferred from 1,446 aligned nucleotide sites of 16S rRNA gene sequences is shown with bootstrap values of no less than 70%. The gut symbionts acquired from the soil are highlighted in bold type, while the gut symbionts of field-collected *M. japonensis* adults are colored. The sample IDs are listed in Table S1 and Table S2. The arrow indicates the gut symbiont of an adult that emerged in the sterile water treatment

In all adult insects from the symbiont-suspended water treatment, the 16S rRNA gene sequences of their gut symbionts were identical to those of the experimentally supplied bacterial isolate from ITSM1. In adult insects from the field-collected soil treatment, the 16S rRNA gene sequences of their gut symbionts exhibited variation, but phylogenetic analysis revealed that most of them (18 of 19) belonged to groups 1, 3, or 4 (Fig. 4). Only one adult insect (sample ID SOIL13) was associated with a gut symbiotic bacterium that did not fall into any of the groups 1–6, and BLAST searches with the 16S rRNA gene sequence as a query retrieved sequences of the Enterobacteriaceae, with the top hit being a *Leclercia* species isolated from human puncture fluid (GenBank accession number CP049786; 99.6% sequence identity) (Fig. 4). In an adult insect from the sterile water treatment group, a 16S rRNA sequence belonging to group 1 was detected in the midgut fourth section (Fig. 4), probably due to accidental contamination.

## Discussion

This study demonstrated that the midgut symbiotic bacteria of *M. japonensis* are not vertically transmitted, but are environmentally acquired by the host nymphs in each generation. This is the first reported case of environmental symbiont acquisition in stinkbugs of the pentatomoid family Cydnidae, wherein vertical symbiont transmission has been reported in four other species [14, 27, 47]. We showed that the symbiotic bacteria of field-collected *M. japonensis* adults exhibit remarkably high diversity, forming six distinct phylogenetic groups, and most of the symbionts are cultivable and closely related to free-living *Pantoea*-allied bacteria (Fig. 2 and Fig. S3). The symbiont cultivability shown in this study, 86.1% of the symbionts, is inherently underestimated because we used only LB medium and limited the incubation time to 24 h. These results strongly suggest that the symbiotic bacteria of *M. japonensis* are acquired from the environment. Furthermore, we showed that symbiont phylogenetic groups did not reflect the host phylogeny (Fig. S4a), and bacteria were rarely detected on the egg surface (Fig. S5), suggesting the absence of vertical symbiont transmission. The rearing experiment confirmed environmental symbiont acquisition: newly hatched nymphs supplied with a symbiont source (symbiont-suspended water or field-collected soil) acquired symbiotic bacteria and reached adulthood, whereas those deprived of such a symbiont source became aposymbiotic and died at the first instar (Figs. 3 and 4). To our knowledge, subsocial behavior in which mothers and offspring live together has not been reported in any *Macroscytus* species, nor did we observe such behavior in our *M. japonensis* rearing system. Therefore, vertical symbiont transmission via coprophagy, as reported in the subsocial cydnid species *C. atterimus* [14], is unlikely in *M. japonensis*. The phylogenetic relationship between the genus *Macroscytus* and other cydnid genera in which vertical symbiont transmission has been reported, such as *Cydnus*, *Adomerus* and *Canthophorus* [14, 27, 47]*,* is of evolutionary interest but is currently unresolved [64].

The saw-toothed stinkbug *Me. gracilicorne*, a member of the pentatomoid family Dinidoridae, has been shown to acquire γ-proteobacterial gut symbionts from the environment in each generation [43]. Interestingly, *M. japonensis* and *Me. gracilicorne*, which belong to different genera, both acquire symbionts environmentally and share symbionts of groups 1, 4, and 6 (Fig. 2). In both *M. japonensis* (Fig. 2 and Fig. S3) and *Me. Gracilicorne* [43], group 1 symbiont infections are most prevalent. In addition, *M. japonensis* shares group 2 symbionts with the cydnid burrower bug *Adrisa magna*, whose symbiont transmission mode is currently unknown (Fig. 2 and Fig. S3). We expect that future studies will discover more pentatomoid species that acquire symbionts environmentally, especially from Cydnidae and Dinidoridae, and that these species may share symbiont groups with *M. japonensis* and *Me. gracilicorne*. Furthermore, the fact that vertically transmitted symbionts of pentatomid and scutellerid stinkbugs are included in the groups 1, 3, 4, and 6 (Fig. 2) [35, 38, 57] is also of great interest in the context of the evolution of symbiont transmission modes.

Given the vast diversity of microorganisms in the soil environment [65], it is likely that *M. japonensis* nymphs orally ingest diverse microorganisms, from which only symbiotic bacteria are selected to establish a specific infection in the crypts of the midgut fourth section. Although the mechanisms of symbiont selection in *M. japonensis* remain unknown, it has been documented that a specific constricted region between the third and fourth sections of the midgut is involved in symbiont sorting in the coreoid stinkbug *Riptortus pedestris*, in which nymphs acquire β-proteobacterial symbionts environmentally [66]. As in *R. pedestris* and other pentatomomorphan stinkbugs, the midgut of *M. japonensis* consists of four morphologically distinct sections [11, 56]. Therefore, symbiont sorting in the constricted region between the third and fourth sections of the midgut seems plausible in *M. japonensis* but requires further investigation. The results of nymphal incubation with field-collected soil (Fig. 4) suggest that bacteria belonging not only to *Pantoea* but also to related genera, including *Enterobacter* and *Leclercia,* may be able to establish infections in the midgut crypts of *M. japonensis*.

In the Japanese populations of *M. japonensis*, group 1 symbiont infections were most prevalent (63.9%), followed by group 3 infections (20.8%), with the remainder being minor (< 7%) (Fig. 2 and Fig. S3). Interestingly, nymphal incubation with field-collected soil resulted in similar symbiont infection frequencies in adult insects (Fig. 4). Several factors may account for these symbiont infection frequencies. Group 1 and 3 symbionts may be more favored by symbiont selection in *M. japonensis* than symbionts of the other groups. It is possible that the ability to colonize the midgut and/or fitness effects of group 1 and 3 symbionts are superior to those of other symbionts. Colonization competitiveness between symbionts can also influence the frequency of symbiont infections in host populations [45, 67]. Furthermore, group 1 and 3 symbionts may be more abundant than other symbiont groups in the soil of *M. japonensis* field habitats. In this context, the soil microbiota in *M. japonensis* field habitats is expected to differ between the Japanese mainland and southwestern island populations, where group 1 and 3 symbiont infections are most prevalent, respectively (Fig. S4b). These hypotheses are experimentally testable and should be verified in future studies.

The rearing experiment revealed that all nymphs deprived of symbiont sources failed to reach the second instar and died at the first instar (Fig. 3), indicating the essential nature of gut symbiotic bacteria for the growth and survival of *M. japonensis* nymphs. Genomic and physiological studies have revealed the provision of essential amino acids and vitamins by gut symbionts in plant sap-sucking stinkbugs of the pentatomoid families Plataspidae and Urostylididae [31, 68, 69]. Both adults and nymphs of *M. japonensis* feed on seeds of various plants, including the wild cherry tree *Prunus jamasakura* (Rosaceae), the camphor tree *Camphora officinarum* (Lauraceae) and the kurogane holly *Ilex rotunda* (Aquifoliaceae) [62]. As these foods are likely protein-rich, it is conceivable that the gut symbionts of *M. japonensis* may provide the host with essential B vitamins for host growth and survival, as has been demonstrated in the seed-sucking cotton stainer bug *Dysdercus fasciatus* [70]. Future genomic and genetic studies of the cultivable symbionts may elucidate the physiological aspects of the *M. japonensis*-gut bacteria symbiosis. Whether there are differences in symbiont physiological functions between symbiont groups or strains is another question that should be investigated in future studies.

The results of the rearing experiment (Fig. 3) also indicate that *M. japonensis* nymphs acquire gut symbionts during the first instar. This is in contrast to a report from the coreoid stinkbug *R. pedestris*, in which symbiont acquisition from the environment occurs mainly in the second instar, because the midgut fourth section of first-instar nymphs is atrophied and unsusceptible to symbiont infection [48]. In pentatomoid stinkbugs in which gut symbionts are vertically transmitted, first-instar nymphs ingest gut symbionts upon hatching [5] and promptly establish symbiont infection of the midgut fourth section [71]. Therefore, the midgut fourth section of first-instar nymphs of *M. japonensis*, a member of the Pentatomoidea, is also likely to be susceptible to symbiont infection. Recently, second-instar nymphs of the coreoid stinkbug *Anasa tristis* have been shown to exhibit active searching behavior to acquire β-proteobacterial symbionts from the environment [72]. Whether first-instar nymphs of *M. japonensis* exhibit such behavior is currently unknown. First-instar nymphs of *M. japonensis* appear to be less mobile than second-instar nymphs of coreoid stinkbugs and are unlikely to be able to move over large areas. Therefore, whether first-instar nymphs of *M. japonensis* are able to acquire symbionts and which group of symbionts they acquire are likely to depend more on where their mothers oviposit than on nymphal behavior. The possibility that oviposition site selection by *M. japonensis* females may influence symbiont acquisition by their offspring also merits future study.

## Conclusions

In conclusion, the gut symbiotic bacteria of the cydnid burrower bug *M. japonensis* are environmentally acquired rather than being vertically transmitted. This study highlights Cydnidae as the only pentatomoid family that includes species that environmentally acquire symbionts and those that vertically transmit symbionts, providing an ideal platform for comparative studies of the ecological and environmental factors that influence the evolution of symbiont transmission modes. Future studies should aim to comprehensively investigate symbiont transmission modes in cydnid genera and species as well as their phylogenetic relationships.

### Supplementary Information

Additional file 1: Table S1. Insect samples, symbiont type, symbiont cultivability and nucleotide sequence accession numbers. Table S2. Sample IDs and nucleotide sequence accession numbers of gut symbiotic bacteria acquired from the soil.

Additional file 2: Fig. S1. Collection sites for the *M. japonensis* samples used in this study. Fig. S2. Petri dishes setting for sterile water treatment, symbiont-suspended water treatmentand field-collected soil treatment. Fig. S3. Phylogenetic placement of gut symbiotic bacteria from field-collected *M. japonensis* adults based on *groEL* gene sequences. A maximum likelihood tree inferred from 833 aligned nucleotide sites is shown with bootstrap values of no less than 70%. The gut symbiotic bacteria of *M. japonensis* are colored, and the sample IDs are listed in Table S1. Asterisks denote gut symbiotic bacteria uncultivable on LB agar plates. The gut symbiotic bacteria of the other stinkbugs are highlighted in boldface. An arrow indicates the isolated bacterial strain used in the rearing experiment. Brackets contain accession numbers. Fig. S4.Mapping of symbiont groups on a maximum likelihood phylogeny of *M. japonensis* inferred from 650 aligned nucleotide sites of mitochondrial COI gene sequences. Bootstrap values of no less than 70% are shown. The sample IDs are listed in Table S1, and blue, red, green, orange, brown, and purple indicate infections with symbiont groups 1, 2, 3, 4, 5, and 6, respectively. Arrowheads indicate 18 insect samples collected from the four southwestern island populations.Infection frequencies with group 1–6 symbionts in 19 mainland populations, and four southwestern island populationsof M. japonensis. Fig. S5. Diagnostic PCR detection of bacterial and insect mitochondrial genes in the eggs of *M. japonensis* females. Lanes e1 to e6, DNA extracted from individual eggs; lane M, DNA size markersfrom 100 bp to 1,000 bp in 100-bp increments and 1,500 bp; lane N, negative control; lane P, positive control. Arrows indicate the faint bands of PCR products.

## Data Availability

The newly determined symbiont 16S rRNA, *groEL* and insect mitochondrial *COI* gene sequences were deposited in the DDBJ database with accession numbers LC810670–LC810885 and LC811566–LC811588. Further information should be directed to and will be fulfilled by the corresponding author.
