# Supplementary material for: The burrower bug Macroscytus japonensis (Hemiptera: Cydnidae) acquires obligate symbiotic bacteria from the environment
Source: Zoological Lett. 2024 Aug 2;10:15. doi: 10.1186/s40851-024-00238-9 (PMC11297623; doi:10.1186/s40851-024-00238-9)
Supplement: Supplementary file 2 — Additional file 2: Fig. S1. Collection sites for the M. japonensis samples used in this study. Fig. S2. Petri dishes setting for sterile water treatment, symbiont-suspended water treatmentand field-collected soil treatment. Fig. S3. Phylogenetic placement of gut symbiotic bacteria from field-collected M. japonensis adults based on groEL gene sequences. A maximum likelihood tree inferred from 833 aligned nucleotide sites is shown with bootstrap values of no less than 70%. The gut symbiotic bacteria of M. japonensis are colored, and the sample IDs are listed in Table S1. Asterisks denote gut symbiotic bacteria uncultivable on LB agar plates. The gut symbiotic bacteria of the other stinkbugs are highlighted in boldface. An arrow indicates the isolated bacterial strain used in the rearing experiment. Brackets contain accession numbers. Fig. S4.Mapping of symbiont groups on a maximum likelihood phylogeny of M. japonensis inferred from 650 aligned nucleotide sites of mitochondrial COI gene sequences. Bootstrap values of no less than 70% are shown. The sample IDs are listed in Table S1, and blue, red, green, orange, brown, and purple indicate infections with symbiont groups 1, 2, 3, 4, 5, and 6, respectively. Arrowheads indicate 18 insect samples collected from the four southwestern island populations.Infection frequencies with group 1–6 symbionts in 19 mainland populations, and four southwestern island populationsof M. japonensis. Fig. S5. Diagnostic PCR detection of bacterial and insect mitochondrial genes in the eggs of M. japonensis females. Lanes e1 to e6, DNA extracted from individual eggs; lane M, DNA size markersfrom 100 bp to 1,000 bp in 100-bp increments and 1,500 bp; lane N, negative control; lane P, positive control. Arrows indicate the faint bands of PCR products. [file 40851_2024_238_MOESM2_ESM.pdf]

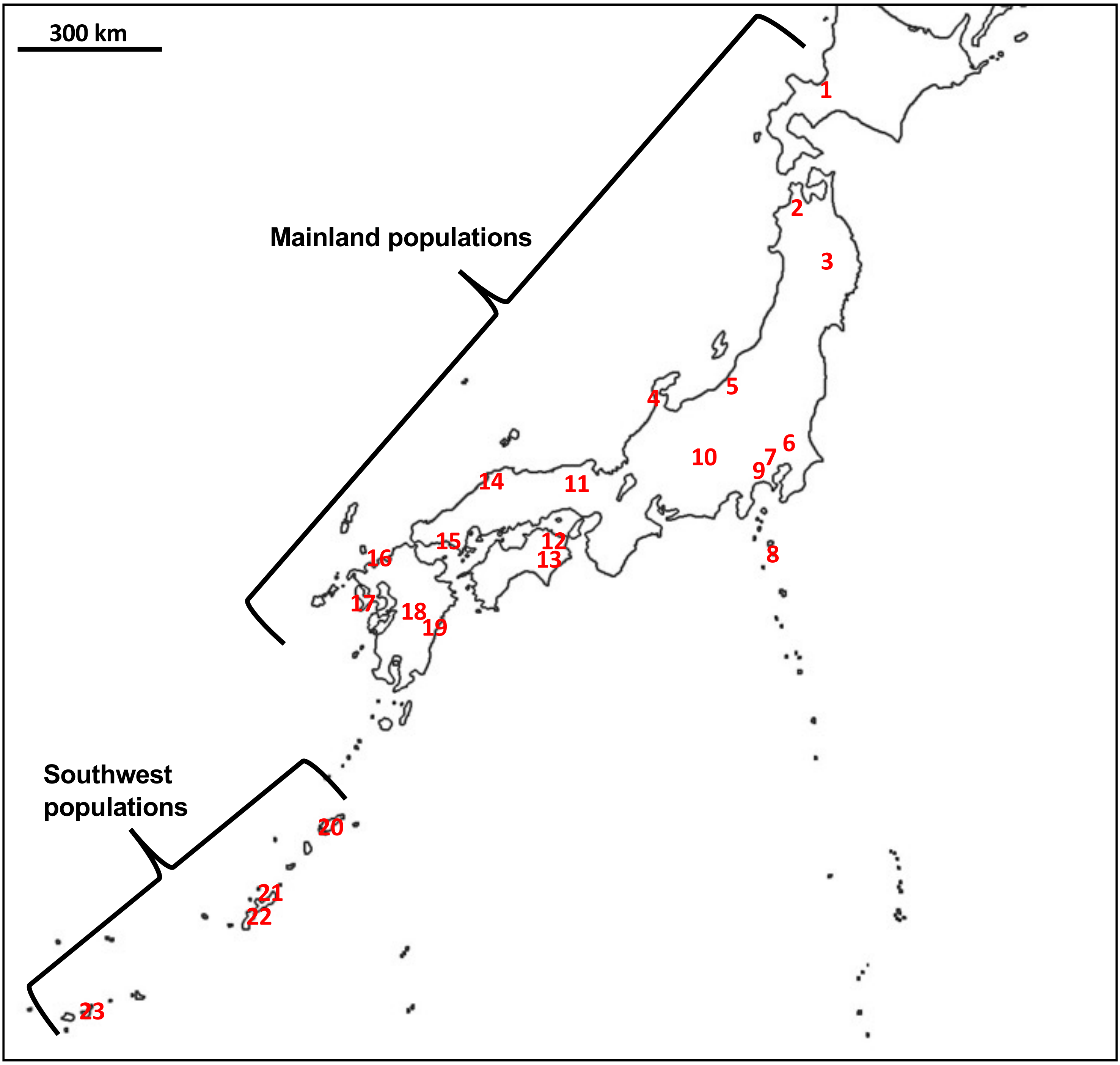

1	Sapporo, Hokkaido	13	Katsuura, Tokushima
2	Hirosaki, Aomori	14	Izumo, Shimane
3	Morioka, Iwate	15	Hikari, Yamaguchi
4	Hodatsu-Shimizu, Ishikawa	16	Itoshima, Fukuoka
5	Nagaoka, Niigata	17	Nagayo, Nagasaki
6	Tsukuba, Ibaraki	18	Hinokage, Miyazaki
7	Tokyo, Tokyo	19	Nobeoka, Miyazaki
8	Mikura Is., Tokyo	20	Amami-Oshima Is., Kagoshima
9	Kawasaki, Kanagawa	21	Kunigami, Okinawa
10	Minami-Minowa, Nagano	22	Nakagusuku, Okinawa
11	Tamba, Hyogo	23	Ishigaki Is., Okinawa
12	Naruto, Tokushima		

Fig. S1. Collection sites for the *M. japonensis* samples used in this study.

Fig. S1

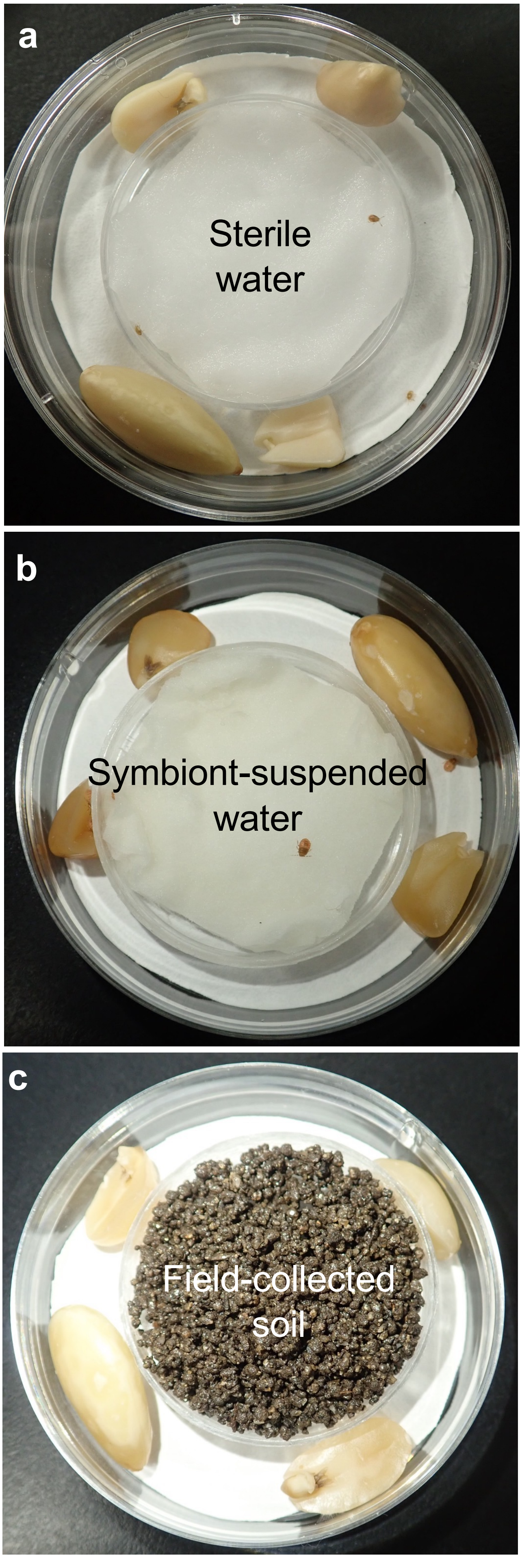

Fig. S2. Petri dishes setting for sterile water treatment (a), symbiont-suspended water treatment (b) and field-collected soil treatment (c).

Fig. S2

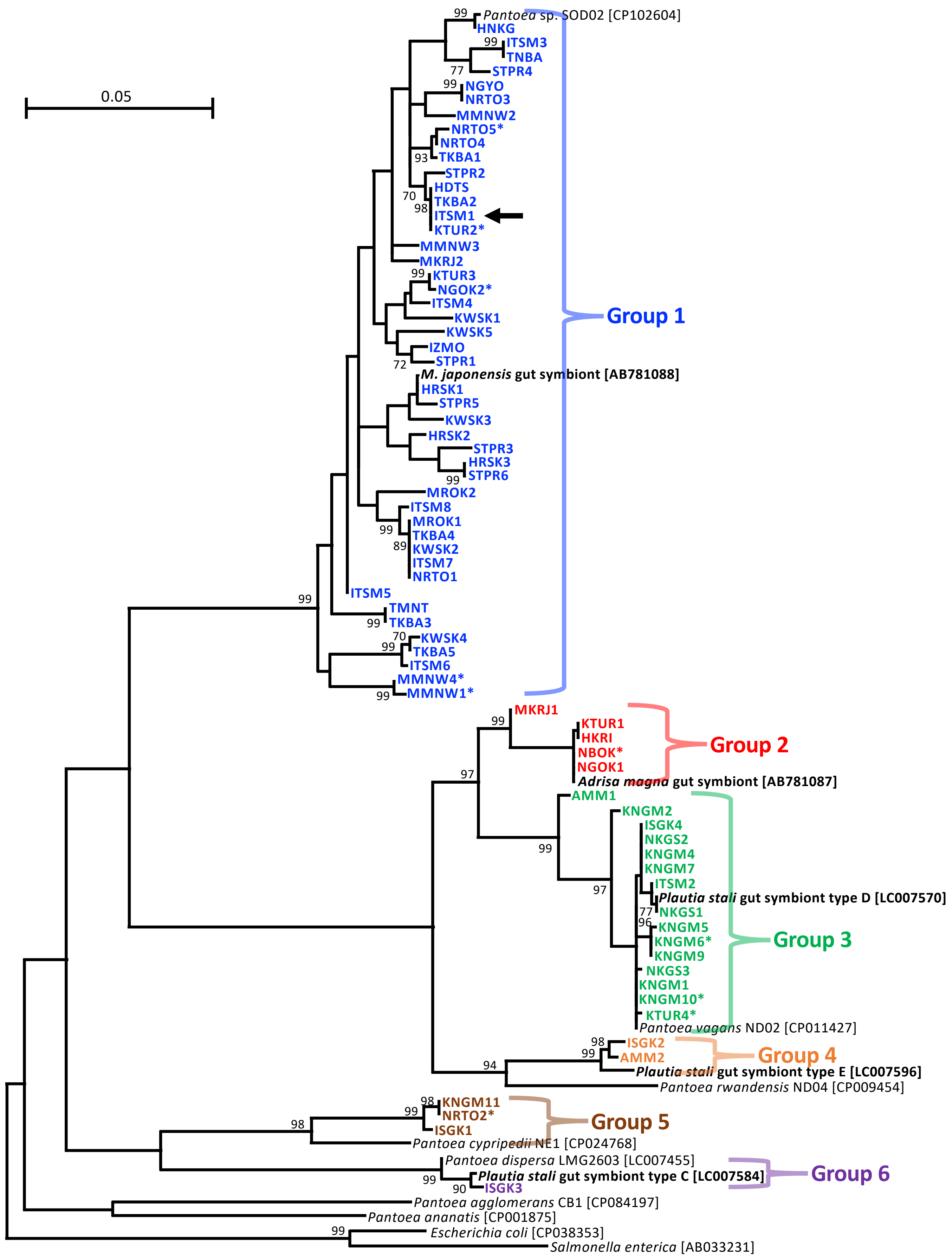

Fig. S3. Phylogenetic placement of gut symbiotic bacteria from field-collected *M. japonensis* adults based on *groEL* gene sequences. A maximum likelihood tree inferred from 833 aligned nucleotide sites is shown with bootstrap values of no less than 70%. The gut symbiotic bacteria of *M. japonensis* are colored, and the sample IDs are listed in Table S1. Asterisks denote gut symbiotic bacteria uncultivable on LB agar plates. The gut symbiotic bacteria of the other stinkbugs are highlighted in boldface. An arrow indicates the isolated bacterial strain used in the rearing experiment. Brackets contain accession numbers.

Fig. S3

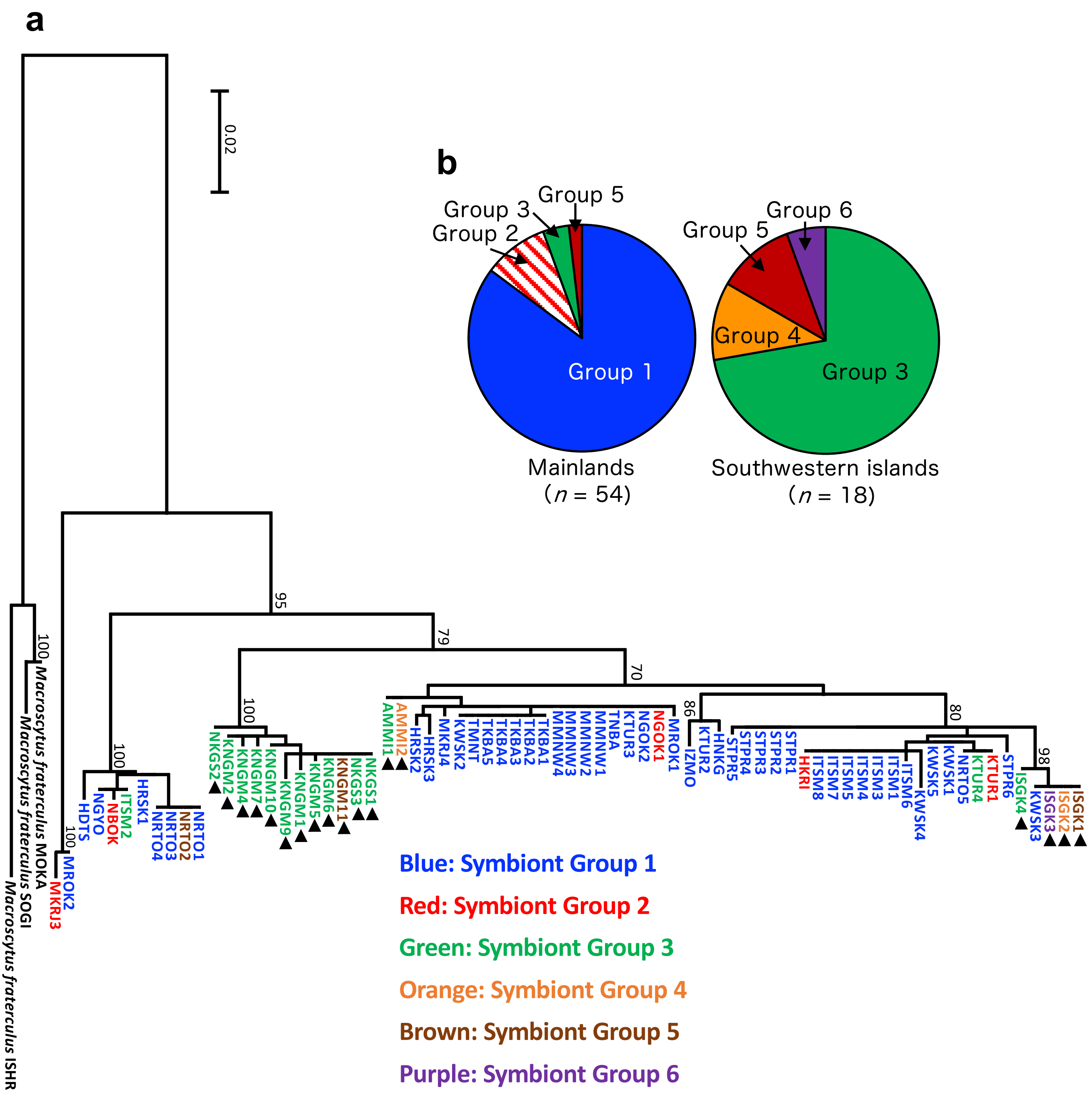

Figure S4. (a) Mapping of symbiont groups on a maximum likelihood phylogeny of *M. japonensis* inferred from 650 aligned nucleotide sites of mitochondrial *COI* gene sequences. Bootstrap values of no less than 70% are shown. The sample IDs are listed in Table S1, and blue, red, green, orange, brown, and purple indicate infections with symbiont groups 1, 2, 3, 4, 5, and 6, respectively (see Fig. 2 and Fig. S3). Arrowheads indicate 18 insect samples collected from the four southwestern island populations. (b) Infection frequencies with group 1–6 symbionts in 19 mainland populations (sites 1–19 in Fig. S1), and four southwestern island populations (sites 20–23 in Fig. S1) of *M. japonensis*.

Fig. S4

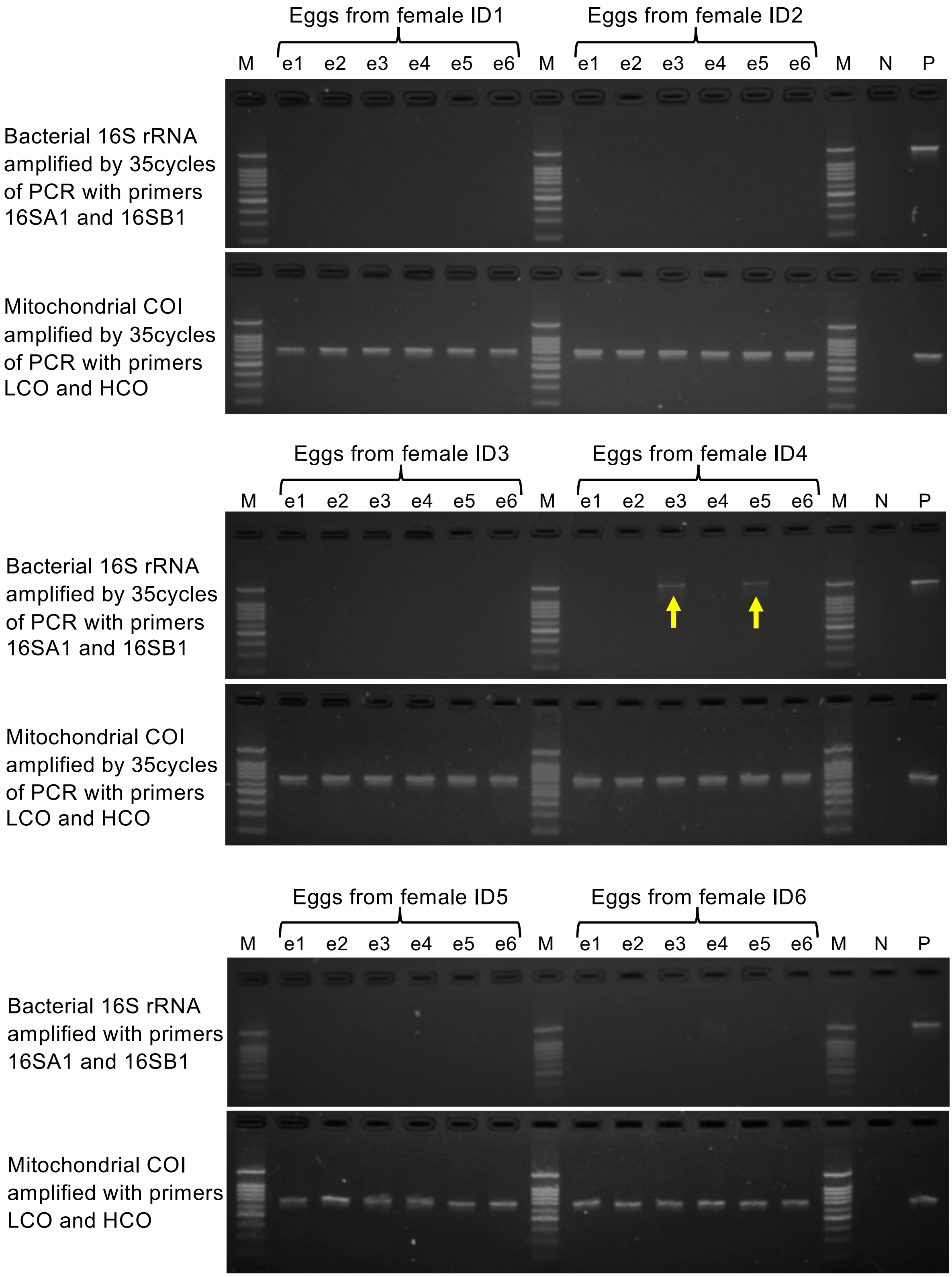

Figure S5. Diagnostic PCR detection of bacterial and insect mitochondrial genes in the eggs of *M. japonensis* females. Lanes e1 to e6, DNA extracted from individual eggs; lane M, DNA size markers (from bottom to top) from 100 bp to 1,000 bp in 100-bp increments and 1,500 bp; lane N, negative control; lane P, positive control. Arrows indicate the faint bands of PCR products.

Fig. S5
